# The Double-Edged Sword Property of Mesenchymal Stem Cell-Derived Exosomal microRNAs in Colorectal Cancer

**DOI:** 10.5152/tjg.2024.23541

**Published:** 2024-10-01

**Authors:** Hongliang Yao, Caihua Sun, Chengjun Wang, Jipan Liu, Yun Li, Li Li, Bin Zhao, Jia Liu

**Affiliations:** 1 The Second Department of General Surgery, Hengshui People’s Hospital, Hengshui, Hebei Province, China

**Keywords:** Colorectal cancer, exosome, mesenchymal stem cells, microRNA

## Abstract

Mesenchymal stem cells (MSCs)-based therapies are promising therapeutic strategies for cancer treatment, because of their strong immunomodulatory and tissue regeneration abilities. In case of colorectal cancer (CRC), MSCs indicate a double-edged sword activity. Some reports declared the inhibitory effects of MSCs on the proliferation, migration, and infiltration of cancer cells to suppress the CRC initiation and development, whereas others showed the tumor-promoter impacts of MSCs on the progression of CRC. Recent investigations have revealed that exosomal microRNAs (Exo-miRs) derived from MSCs (MSCs-Exo-miRs) are attributed to such paradoxical effect. Thus, the current review aimed to seek the role of MSCs-Exo-miRs in CRC progression and their therapeutic potential for the CRC treatment.

**Main Points**

- Exosomal microRNAs (Exo-miRs) derived from various mesenchymal stem cells (MSCs) can act as an oncomiR or a tumor suppressor miR in colorectal cancer (CRC).
- Human umbilical mesenchymal stem cells (hUMSC)-Exos can suppress the progression of CRC by delivering tumor-suppressive miRs, including miR-431-5p and miR-3940-5p.
- Bone marrow mesenchymal stem cell (BMSC)-Exos can suppress the progression of CRC by delivering tumor-suppressive miRs, including miR-22-3p, miR-16-5p, and miR-4461.
- Human bone marrow mesenchymal stem cells (hBMSCs) secrete exosomes containing oncomiR-424, which promotes the CRC progression.
- Human colorectal cancer (hCRC)-MSCs can secrete exosomes that induce CRC progression by delivering concomiRs, including miR-222 and miR-30a.

## Introduction

Colorectal cancer (CRC), as a malignancy beginning from the rectum and/or colon, is the third most common cancer in terms of diagnosis (6.1%) and second in terms of cancer-related deaths (9.2%). Rectal and colon cancers constitute approximately 28% and 72% of all CRC incidences respectively. It is estimated that the overall numbers of rectal and colon cancer-caused mortality will elevate to about 71% and 60% respectively, by the year 2035.^1^

Various risk factors are involved in CRC progression, including non-modifiable and modifiable risk factors. Notably, individuals cannot control non-modifiable risk factors. These include family history as well as personal and medical history, such as ethnicity, race, age, sex, history of inflammatory bowel disease, adenomatous polyps history, cholecystectomy, diabetes, and gut microbiome. On the other hand, modifiable risk factors include environmental factors and lifestyles or individual habits, such as physical inactivity, obesity, overweight, alcohol consumption, cigarette smoking, and unsuitable dietary habits.^2^ Near to 96% of CRC incidences present as adenocarcinoma, which stems from the secretory epithelial cells covering the surface of the inner cavity of the rectum and/or colon. Indeed, CRC progresses once these cells acquire a series of epigenetic or genetic changes inducing excessive proliferation.^2^ These hyperproliferative cells develop into a benign adenoma called polyps, which eventually advance to invasive carcinoma termed adenocarcinoma and migrate to various distant tissues through blood and lymphatic arteries.^2^ Same as other cancers, CRC is classified from stage 0 to stage IV. Excessive proliferation leads to adenoma or polyps (a benign tumor), forming stage 0. Close to 10% of adenomatous polyps may develop into adenocarcinoma invading the muscularis propria (stage I). At stages II and III, the tumor volume is elevated and accordingly invades the serosa and visceral peritoneum respectively. Eventually, at stage IV, it will metastasize through blood or lymphatic vessels.^2^ Of note, the stage of CRC determines the disease’s severity and the suitable approaches for CRC treatment. For stages 0-II CRC, the standard therapeutic option is surgery. For stage III CRC, surgery and adjuvant chemotherapy, and for stage IV and recurrent CRC, surgery, chemotherapy, and targeted therapy are preferred.^2^ Despite available therapeutic options, there is no fully effective treatment approach yet.^3,4^

The first lines of treatment for CRC are surgery, chemotherapy, radiotherapy, and adjuvant therapy. Principally, radiotherapy or chemotherapy is administered before or after surgery to stabilize the tumor or assist in shrinkage. Currently, the prominent chemotherapy regimens include monotherapy, such as treatment with fluoropyrimidines like 5-Fluorouracil (5-FU), and combined-drug regimens, including Capecitabine (XEL or CAP or XELODA), Irinotecan (IRI), and Oxaliplatin (OX). The main chemotherapy regimens in the first-line treatment include combination therapies such as CAPIRI (CAP + OX), XELOX or CAPOX (CAP + OX), FOXFIRI (5-FU + IRI), and FOLFOX (5-FU + OX).^5^ Notably, these chemotherapy regimens exert various irreversible side effects, such as low tumor-specific selectivity, variable innate and acquired resistance, unsatisfactory response rates, and systemic toxicity.^2^

In recent decades, several novel approaches have been established to refine or replace standard CRC chemotherapy. Currently, there are different alternative FDA-approved therapeutic approaches for CRC treatment in the clinic, including immunotherapy using monoclonal antibodies (mAbs) such as the anti-angiogenesis drug bevacizumab, the anti-epidermal growth factor receptor (EGFR) drug cetuximab, and checkpoint inhibitors such as the CTLA-4 (cytotoxic T lymphocyte antigen 4) inhibitor ipilimumab and PD-1 (programmed cell death protein 1) inhibitors pembrolizumab and nivolumab.^5^ In addition, several approaches are being developed in preclinical and clinical studies, including cancer vaccines stimulating antigen-specific B- or T-cell activity against cancer, such as DC vaccine and oncoVAX,^6^ as well as cell therapy.^7^

Mesenchymal stem cell (MSC)-based therapies are promising therapeutic strategies to treat cancer because of their strong immunomodulatory and tissue regeneration abilities. In preclinical studies, MSCs have been used as cell therapy or cell-free isolated therapies, such as exosomes. Notably, due to some limitations and possible side effects with cell therapy, recent experimental studies have focused on exosome therapies with higher beneficial effects, without limitations and side-effects.^8^ Of note, the therapeutic potential of exosomes is attributed to functional cargoes such as microRNAs (miRs). As described in the next sections, several experimental investigations have demonstrated that exosomal miRs (Exo-miRs) isolated from different types of MSCs can provide therapeutic tools or targets. Their overexpression or inhibition respectively, suppresses CRC progression. This current review aimed to explore the recent preclinical advances on Exo-miRs derived from MSCs (MSCs-Exo-miRs) for CRC treatment. To this end, we initially provide a general survey on the therapeutic activities and limitations of MSCs for cancer treatment throughout the next subsection. Afterwards, the therapeutic impacts of MSCs-Exo-miRs on CRC progression will be discussed in the final sections.

## Role of Mesenchymal Stem Cells in Colorectal Cancer

Mesenchymal stem cells are adult stem cells that are found and isolated from various tissues and organs such as bone marrow, the circulating blood, umbilical cord, placenta, teeth, hair follicles, muscle, adipose tissue, as well as cancerous tissues. These are self-renewal and pluripotent cells with the ability of multi-lineage differentiation, rapidly proliferating and transforming into various specialized cell types, such as chondrocytes, adipocytes, osteocytes, and monocytes. Mesenchymal stem cells isolated from various sources show various potentials for proliferation, differentiation, and migration, which determine their capacity for anti-cancer therapy. Mesenchymal stem cells therapy, which includes all strategies using MSCs, has proven to be a strong fighter against tumor cells. Mesenchymal stem cells possess specific biological activities and have been broadly employed to assist other treatments or to transport therapeutic mediators in treating a wide range of tumors. Mesenchymal stem cells therapy can elevate the therapeutic efficacy of other therapies due to its specific targeting of cancer cells, thus decreasing off-target events. Several MSCs-based strategies are being studied in preclinical investigations, revealing both benefits and challenges for tumor therapy. Mechanistically, both cancer cells and cancer-related immune cells can provoke this process by secreting different chemoattractant mediators. For instance, IL-6, CCL-25, SDF-1, and CXCL16 secreted by breast cancer, multiple myeloma, osteosarcoma, and prostate cancer cells promote MSC migration toward the tumor microenvironment respectively.^9^ Further, cancer-related immune cells secrete pro-inflammatory cytokines IL-1β and TNF-α that exert critical roles in the migration toward and differentiation within the tumor microenvironment.^10^

Importantly, MSC transplantation can improve the post-treatment complications of cancers, such as severe damage to normal tissues and the hematopoietic system due to high-dose therapy and invasive tumor removal. Moreover, MSCs with immunomodulatory abilities could effectively decrease undesirable immune responses in patients with refractory “graft-versus-host disease” (GVHD). Of note, a multi-center clinical trial (NCT02923375) showed the efficacy, tolerability, and safety of MSC infusion in patients who have steroid-resistant GVHD. Mesenchymal stem cells could accelerate the recovery of damaged tissues/organs and improve body tolerance to high-dose chemotherapy, thus enhancing tumor-killing impacts.^11^

In the case of CRC, MSCs indicate a double-edged sword activity. Some reports have declared the inhibitory effects of MSCs on the proliferation, migration, and infiltration of cancer cells to suppress CRC initiation and development, whereas others have shown the tumor-promoter impacts of MSCs on the progression of CRC. It was shown that the administration could inhibit tumorigenesis in IBD mice by suppressing the expression of proinflammatory cytokines (IL-1β, IL-6, and TNF-α) and activation of STAT3.^12^ Another preclinical study indicated that MSCs could exert antitumor activities by promoting apoptosis via inhibiting the cell cycle in the G1 phase and also by dysregulating the Wnt and TGF-β-Smad signaling pathways.^13^ In another study, low doses of X-ray irradiation and ultraviolet radiation promoted BM-MSCs to produce cancer-suppressing cytokines IFN-γ and TNF-α to inhibit CRC cell proliferation and induce apoptosis.^14^ Further, MSCs were also reported to regulate the immune responses in the tumor microenvironment by modulating the profile of cytokine secretion by immune cells such as T cells.^15^ Anti-tumor impacts were further supported by clinical investigations that showed MSCs could elevate the survival time of patients who exhibited tumor metastasis and complications resulting from CRC.^16^ In addition, MSC therapy was demonstrated to prevent CRC progression in UC patients by inhibiting chronic inflammatory responses as well as inducing a chemoprophylaxis impact.^17^

On the other hand, there are also reports that show tumor-promoter impacts of MSCs in CRC. Of note, MSCs show tumor-tropic ability whereby they directly migrate toward the tumor microenvironment and create a key component of the tumor stroma, where they can differentiate into myofibroblasts or endothelial cells, which participate in the regulation of stem cell maintenance, epithelial proliferation, intestinal inflammation, angiogenesis, as well as extracellular matrix remodeling and metastasis.^18^ Supporting this, it was shown that MSCs could exert tumor-promoter activity in CRC through changing the expression of cyclin and suppressing apoptosis via the AMPK/mTOR-mediated activation of NF-κB.^19^ Consistently, another study indicated that MSCs derived from human CRC could induce CRC progression through IL-6/JAK2/STAT3 signaling and activating PI3K/AKT signaling.^20^ Further, it was shown that TGF-β1-treated MSCs could differentiate into cancer-associated fibroblasts and induce migration and invasion of CRC cell lines HT29 and HCT116 cells through activating the JAK/STAT3 signaling cascade.^21^ Another study demonstrated that MSCs could induce the proliferation, tumorigenesis, and invasion of CRC cells by secreting soluble NRG1 and activating the HER2/HER3-dependent PI3K/AKT signaling pathway in CRC cells.^22^ Moreover, inflammation-activated human MSCs were found to induce the epithelial–mesenchymal transition (EMT) process and the tumor-stromal formation in CRC through the CCL5/β-catenin/Slug pathway.^23^ and producing secreted protein acidic and rich in cysteine (SPARC).^24^ In addition, the CXCR4/TGF-β1 axis was also found to mediate the differentiation of MSCs to CAFs, which transform the tumor microenvironment, thereby inducing CRC growth and metastasis.^25^ These findings can be further supported by in vivo studies that indicated MSCs could promote tumor growth through interacting with CRC cells via CCL3/4/5-CCR5.^26^

Besides, there is also growing evidence that declares MSCs perform their activities via secreting various paracrine mediators, such as exosomes that act as important regulators of cell-to-cell communications via their miRs content, which may influence tumor progression, survival, as well as metastasis.

## Role of Mesenchymal Stem Cell-derived Exo-miRs in the Colorectal Cancer Progression

Exosomes include an important sub-class of lipid bilayer extracellular vesicles generated through the exocytosis process of the intracellular multivesicular bodies via a wide range of cell types such as MSCs. Exosomes mirror the molecular composition of origin cells and incorporate in the intercellular communication between distant and/or neighboring cells. Exosome-dependent cell cross-talk emanates from its ability to deliver biological data from donor cells to recipient cells. The exosome composition is mainly dependent on the biological properties of parental cells, determining the exosomal activity. Some cargoes are present in almost all exosomes, and others are specific to a cell type and/or a tissue. Overall, exosomes contain different biological molecules, such as DNA, proteins, mRNAs, and miRs that determine the biological activities of exosomes.^27-30^

**Figure 1.**
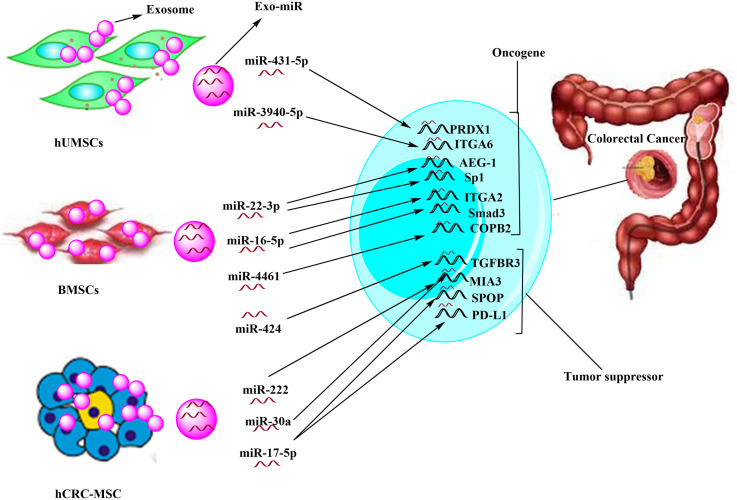
Exosomal miRs derived from various sources of mesenchymal stem cells exerting tumor suppressive or oncogenic effects.

Exosomes produced by MSCs are known to not only recapitulate the biological functions of parent cells but also exhibit superior properties.^27^ Exosomes have the potential to resolve challenges regarding stem cell therapy, such as poor engraftment and low viability, risk of differentiation into unwanted cells and progression to ectopic tissues, possibility of genetic alterations, as well as safety and ethical issues.^27^ Importantly, these vesicles show targeting ability, whereby homing to a particular injured tissue.^27^ They can be taken up by target cells in a cell type-specific manner that occurs via interaction between surface ligands/receptors of exosomes with the recognized cells. There are growing findings that show MSCs-derived exosomes, similar to MSCs, can show a double-edged activity on CRC progression. As discussed in the following sections, Exo-miRs derived from various MSCs acting as an oncomiR or a tumor suppressor miR can mediate this dual effect (Table 1 and Figure 1).

### Role of human Umbilical Mesenchymal Stem Cell-Derived Exo-miRs in the Colorectal Cancer Progression

Experimental studies indicated that the administration of human umbilical mesenchymal stem cells (hUMSCs) can suppress the progression of the CRC.^17^ Further studies revealed that exosomes derived from hUMSC exert inhibitory impacts on CRC progression.^31,32^ Of note, it has been shown that the inhibitory impact of hUMSC-Exos is mediated by tumor-suppressive miRs.

It was reported that hUMSCs can deliver miR-431-5p to CRC LoVo cells in vitro, where it inhibited the malignant ability of CRC cells by targeting and suppressing the expression of PRDX1.^32^ The expression of miR-431-5p was detected to be markedly downregulated in CRC tissues and cells,^32-34^ which was associated with the poor prognosis of CRC patients.^32^ Importantly, miR-431-5p expression was found to be correlated with LNM (lymphatic node metastasis), TNM (tumor, node, and metastasis), and differentiation of CRC patients.^32^ Besides, the ectopic expression of miR-431-5p was found to reduce the proliferation, invasion, and migration of CRC cells, whereas the suppressed miR-431 reversed alterations in CRC cells.^32-34^

The other study showed that exosomes from hUMSCs delivered high levels of miR-3940-5p into CRC cells, where it directly suppressed the expression of integrin alpha6 (ITGA6), thus inhibiting epithelial–mesenchymal transition and invasion of CRC cells in vitro and suppressing the cancer progression and metastasis in vivo.^35^ In mechanism, ITGA6 participates in cancer growth and metastasis by modulating the tumor microenvironment and protecting tumor cells.^35^

To sum up, hUMSC-Exos can suppress the progression of CRC by delivering tumor-suppressive miRs, including miR-431-5p and miR-3940-5p. Therefore, these miRs can serve as potential targets for treating or diagnosing CRC.

### Role of Bone Marrow Stem Cell-Derived Exo-miRs in the Colorectal Cancer Progression

Primary reports demonstrated that the bone marrow mesenchymal stem cells (BMSCs) administration could significantly ameliorate the CRC progression in rat and mouse models.^12,36,37^ There is growing evidence that shows the tumor-suppressive Exo-miRs derived from BMSCs mediate the inhibitory effects of BMSCs on the CRC progression.

Of note, a study declared that Exo-miR-22-3p derived from human BMSCs (hBMSCs) can inhibit proliferation and invasion of CRC cells by modulating the activity of the RAP2B/PI3K/AKT pathway in the SW480 cell line.^38^ The tumor-suppressive impact of hBMSC-derived Exo-miR-22-3p has also been also reported by other studies that showed miR-22-3p-enriched hBMSC-Exo can suppress the development of non-small cell lung cancer (NSCLC).^39^ and hepatocellular carcinoma^40^ by downregulating the expression of astrocyte-elevated gene-1 (AEG-1) and specificity protein 1 (Sp1).

The tumor-suppressive effect of BMSC-derived Exo-miRs on CRC was further supported by another in vitro study that revealed miR-16-5p-enriched BMSC-Exo inhibited proliferation, migration, and invasion, while promoting the CRC cell apoptosis by targeting and inhibiting the expression of ITGA2.^41^ In vivo studies indicated that the injection of miR-16-5p-enriched BMSC-Exo can reduce the ITGA2 expression in tumor tissues, thereby remarkably decreasing tumor progression and volume in CRC. Further, the tumor-suppressive function of miR-16-5p had already been already reported by other experiments that detected reduced expression of miR-16-5p and increased levels of its oncogenic targets, Smad3 and ITGA2, in the CRC, and other tumor cells such as chordoma, pancreatic cancer, and gastric cancer, which were associated with undesirable survival rate.^41^

In addition, it was also shown that BM-MSC-Exo can deliver miR-4461 to the CRC cells, where it suppressed proliferation, migration, and invasion of CRC cells, HCT116 and SW480 cells, by targeting and suppressing the coatomer protein complex subunit beta 2 (COPB2) expression.^42^ Notably, COPB2 has a critical role in intracellular protein transport and has shown a tumor-promoting role in various cancer types, such as breast cancer.^43^ Overall, reports mentioned earlier show that the beneficial impacts of BMSCs on CRC may be mediated by exosomes containing tumor-suppressive miRs, including miR-22-3p, miR-16-5p, and miR-4461. Thus, above-mentioned BMSC-derived Exo-miRs could be promising targets for the treatment and diagnosis of CRC.

Besides, it was also shown that hBMSCs secrete exosomes containing oncomiRs which enhance CRC progression. miR-424 is an oncomiR that has been detected to be strongly increased in different cancer cells, such as CRC.^44,45^ Notably, hBMSC-derived Exo-miR-424 was found to induce the growth of CRC via directly inhibiting transforming growth factor beta receptor 3 (TGFBR3).^46^ miR-424 was found to be over-expressed, whereas the expression of TGFBR3 was detected to be reduced in CRC tissues and cell lines.^45^ By targeting TGFBR3, BMSC-derived Exo-miR-424 was found to promote the proliferation, migration, and invasion of CRC cells, induce the cell cycle at the G0/G1 phase, and suppress cell apoptosis, whereas blocking miR-424 reversed these changes and suppressed CRC progression.^46^ Of note, the expression level of miR-424 showed a significant association with distant metastasis, LNM and TNM stages, vascular invasion, tumor infiltration depth, and tumor differentiation degree in patients with CRC.^46^

Therefore, hBM-MSC-Exo-miR-142-3p provides the potential therapeutic target whose inhibition can donate a novel therapeutic approach in CRC treatment. Of note, suppressing oncomiR-142-3p in exosomes secreted by hBMSCs may reverse tumor-promoting impacts of these exosomes and restore the therapeutic potential of BMSC-Exos.

### Role of Cancer Stem Cell-Derived Exo-miRs in the Colorectal Cancer Progression

Like other cancers, CRC contains a small population of cancer stem cells (CSCs) that account for an important component of the tumor stroma and are principally responsible for CRC initiation and development, metastasis, recurrence, and drug resistance. Human CRC-derived MSCs (hCRC-MSCs) were found to secrete exosomes that provide effective intercellular communication in the tumor stroma, inducing CRC progression.^47^ The miR profile analysis revealed a significant increase in oncogenic miR-222 and miR-30a in hCRC-MSC-Exos.^47^ Interestingly, hCRC-MSC-secreted Exo-miR-222 and Exo-miR-30a were found to be transported to the CRC cells,^47^ where they directly inhibit the expression of the tumor-suppressive protein melanoma inhibitory activity protein 3 (MIA3) to promote the proliferation, migration, and metastasis ability of colorectal cells.^47,48^ Notably, inhibiting miR-222 and miR-30a was found to reverse the tumor-inducing impact of hCRC-MSC-Exo on colorectal cells.^47^

The other study showed that CRC-MSC-Exos could induce tumor progression and suppress anti-tumor immunity in CRC by delivering miR-17-5p to CRC cells. Mechanistically, CRCSC-derived Exo-miR-17-5p was found to induce tumor progression and malignant behaviors by inhibiting speckle-type POZ protein (SPOP), and suppress anti-tumor immunity in CRC by blocking the expression of the programmed death ligand 1 (PD-L1).^49^

SPOP is an E3 ubiquitin ligase adapter that acts as a tumor suppressor and has been found to exert an important role in CRC progression via mesenchymal–epithelial transition and matrix metalloproteinases. PD-L1 is an effective inducer of immunogenic tumors to escape from host immune responses. Notably, plasma levels of miR-17-5p were already detected to be increased in metastatic PD-L1^+^ melanoma patients, showing a significant association with PD-L1 expression.^50^ The oncogenic impact of miR-17-5p was primarily exhibited and identified to be upregulated in human tumors and promoted vital physiological responses during disease progression.^51^ Notably, circulating miR-17-5p was found to provide an early biomarker for the non-invasive measurement of CRC.^52^ In another study, miR-17-5p was found to accelerate CRC progression.^53^ Similarly, Exo-miR-17-5p derived from cancer-associated fibroblasts was shown to induce malignant phenotypes of CRC.^54^ Further, clinical investigations indicated that over-expressed Exo-miR-17-5p has roles in pathological stages and grades of CRC patients.^55^

In conclusion, reports mentioned earlier suggest that inhibiting the tumorigenic miR-30a, miR-222, and miR-17-5p can provide a novel strategy to block CRC development.

## Conclusion

Overall, the experimental investigations mentioned earlier suggest that MSC-Exo-miRs mediate the double-edged sword role of MSCs in CRC progression. Indeed, some MSC-Exo-miRs exert a suppressive impact on the proliferation, migration, and metastasis of CRC cells, whereas others exert oncogenic impacts on CRC progression. Notably, Exo-miR-431-5p and Exo-miR-3940-5p derived from hUMSCs, as well as Exo-miR-22-3p, Exo-miR-16-5p, and Exo-miR-4461 derived from BMSCs have been found to exert tumor-suppressive effects on CRC progression. Thus, hUMSCs-derived exosomes containing high levels of tumor-suppressive miR-431-5p and miR-3940-5p, as well as BMSCs-derived exosomes containing high levels of tumor-suppressive miR-16-5p, miR-22-3p, and miR-4461, can be further investigated as therapeutic tools for CRC management. However, BMSCs-isolated exosomes containing oncogenic miR-424 could induce tumor progression and metastasis in CRC. Thus, BM-MSC-Exo-miR-142-3p provides a potential therapeutic target, whose suppression could offer a promising therapeutic approach for CRC treatment. Of note, suppressing oncomiR-142-3p in exosomes secreted by BMSCs may reverse tumor-promoting impacts of these exosomes and restore the therapeutic potential of BMSC-Exos. It was also found that oncogenic Exo-miRs, including Exo-miR-30a, miR-17-5p, and Exo-miR-222, could mediate the tumor-promoter impacts of hCRC-MSCs in CRC progression. Thus, inhibiting these tumorigenic miRs in hCRC-MSCs may offer a novel strategy to block CRC development. These findings suggest novel therapeutic targets for CRC treatment; however, further investigations are required to find specific miR inhibitors, such as AMOs (anti-miRNA antisense oligonucleotides), miR sponges, and miR decoys, which, through complementary sequences, bind and sequester specific miRs from their mRNA targets.

## Figures and Tables

**Table 1.** The Impact of Exosomal MicroRNAs Derived from Various Mesenchymal Stem Cells on the Colorectal Cancer Progression

MSCs	Exo-miR	Molecular Target	Impact on CRC Progression	Ref.
hUMSCs	miR-431-5p	PRDX1	Tumor suppressive	(27)
	miR-3940-5p	ITGA6	Tumor suppressive	(35)
BMSCs	miR-22-3p	AEG-1 and Sp1	Tumor suppressive	(33)
	miR-16-5p	ITGA2 and Smad3	Tumor suppressive	(36)
	miR-4461	COPB2	Tumor suppressive	(37)
	miR-424	TGFBR3	Oncogenic	(41)
hCRC-MSC	miR-222 and miR-30a	MIA3	Oncogenic	(42, 43)
	miR-17-5p	SPOP and PD-L1	Oncogenic	(49)

AEG-1, astrocyte-elevated gene 1; BMSCs, bone marrow mesenchymal stem cells; COPB2, coatomer protein complex subunit beta 2; Exo-miRs, exosomal microRNAs; hCRC-MSC, human CRC-derived mesenchymal stem cells; hUMSCs, human umbilical mesenchymal stem cells; ITGA, integrin alpha; MIA3, melanoma inhibitory activity protein 3; MSCs, mesenchymal stem cells; PD-L1, programmed death ligand 1; Sp1, specificity protein 1; SPOP, speckle-type/POZ protein; TGFBR3, transforming growth factor beta receptor 3.
